# Genome-wide association study of four yield-related traits at the R6 stage in soybean

**DOI:** 10.1186/s12863-019-0737-9

**Published:** 2019-03-29

**Authors:** Xiangnan Li, Xiaoli Zhang, Longming Zhu, Yuanpeng Bu, Xinfang Wang, Xing Zhang, Yang Zhou, Xiaoting Wang, Na Guo, Lijuan Qiu, Jinming Zhao, Han Xing

**Affiliations:** 1 National Center for Soybean Improvement/National Key laboratory of Crop Genetics and Germplasm enhancement, Key laboratory of Biology and Genetics and Breeding for Soybean, Ministry of Agriculture, Nanjing Agricultural University, Nanjing, 210095 People’s Republic of China; 2 The National Key Facility for Crop Gene Resources and Genetic Improvement (NFCRI)/Key Lab of Germplasm Utilization (MOA), Institute of Crop Science, Chinese Academy of Agricultural Sciences, Beijing, 100081 People’s Republic of China

**Keywords:** Soybean [*Glycine max* (L.) Merr.], Yield-related traits, R6 stage, GWAS, Quantitative trait locus, Single nucleotide polymorphism (SNP)

## Abstract

**Background:**

The 100-pod fresh weight (PFW), 100-seed fresh weight (SFW), 100-seed dry weight (SDW) and moisture content of fresh seeds (MCFS) at the R6 stage are crucial factors for vegetable soybean yield. However, the genetic basis of yield at the R6 stage remains largely ambiguous in soybean.

**Results:**

To better understand the molecular mechanism underlying yield, we investigated four yield-related traits of 133 soybean landraces in two consecutive years and conducted a genome-wide association study (GWAS) using 82,187 single nucleotide polymorphisms (SNPs). The GWAS results revealed a total of 14, 15, 63 and 48 SNPs for PFW, SFW, SDW and MCFS, respectively. Among these markers, 35 SNPs were repeatedly identified in all evaluated environments (2015, 2016, and the average across the two years), and most co-localized with yield-related QTLs identified in previous studies. AX-90496773 and AX-90460290 were large-effect markers for PFW and MCFS, respectively. The two markers were stably identified in all environments and tagged to linkage disequilibrium (LD) blocks. Six potential candidate genes were predicted in LD blocks; five of them showed significantly different expression levels between the extreme materials with large PFW or MCFS variation at the seed development stage. Therefore, the five genes *Glyma.16g018200*, *Glyma.16g018300*, *Glyma.05g243400*, *Glyma.05g244100* and *Glyma.05g245300* were regarded as candidate genes associated with PFW and MCFS.

**Conclusion:**

These results provide useful information for the development of functional markers and exploration of candidate genes in vegetable soybean high-yield breeding programs.

**Electronic supplementary material:**

The online version of this article (10.1186/s12863-019-0737-9) contains supplementary material, which is available to authorized users.

## Background

Soybean (*Glycine max* (L.) Merr.) is a widely cultivated oil crop worldwide. Soybean seeds are used to supply edible oil and serve as a source of high-quality plant protein [1]. According to different harvest times and uses, soybean crops can be divided into grain or vegetable crops. Vegetable soybean is harvested during the R6 growth stage when the pods are still green and fully filled with seeds [2]. The characteristics of large pods and large grains are important visual qualities of vegetable soybeans [3, 4]. Therefore, yield has long been considered one of the most important traits in vegetable soybean breeding. The vegetable soybean yield is directly determined by yield components, including the number of pods per plant, seeds per pod, fresh seed weight and fresh pod weight. Furthermore, vegetable soybean seeds have a high moisture content of approximately 70.05%, which serves both as a yield component and as an influencing factor of sensory quality [5]. In maize, grain moisture has a higher *h*^*2*^ than does grain yield, and several quantitative trait loci (QTLs) are commonly associated with grain yield and grain moisture [6]. With economic development, the demand for vegetable soybeans has increased, but there are fewer available reports on the yield of vegetable soybean than grain soybean at present. Therefore, dissecting the genetic basis of soybean yield at the R6 stage is necessary and will help to improve the yield potential of vegetable soybean.

Yield-related traits are usually complex quantitative traits influenced by multiple QTLs. Previous studies were conducted to dissect the genetic basis of yield-related traits in biparental populations. Hundreds of QTLs were detected across the whole genome of soybean, many were simultaneously detected in multiple populations [7–13]. These studies demonstrated that the genetic mapping of quantitative traits using genetic linkage maps is an efficient approach for identifying QTLs. Compared with linkage mapping, a genome-wide association study (GWAS) is a more powerful method for dissecting the QTLs underlying agronomically important traits in natural populations with a high density of markers. Natural populations contain more genetic diversity than cross-derived segregating populations, which can be applied directly in GWAS analysis [14]. In addition, GWAS can effectively identify candidate genes that are closely linked to target traits, due to the low level of genomic linkage disequilibrium (LD) [15–18].

At present, association studies have been successfully performed in grain soybean for yield-related traits. For example, 19 SNPs and 5 haplotypes for yield and yield components were identified in a soybean landrace population [19]. For seed size and shape, a total of 59 large-effect QTLs and 31 QTL-by-environment interactions were identified in another study, which were closely related to seed yield and appearance quality [20]. Furthermore, multiple research groups have searched for QTLs related to flowering time and maturity dates that could influence soybean yield [21, 22]. Many of the above QTLs are located in or near QTLs reported in the previously linkage analysis. Based on the QTLs reported to date, several candidate genes have been identified. Gu et al. (2017) proposed SoyWRKY15a as a candidate gene locus for seed size, and differential expression of its orthologous genes *GmWRKY15a* and *GsWRKY15a* in soybean pods was correlated with the seed weight [23]. However, the molecular mechanism underlying yield-related traits in vegetable soybean remains unclear.

In this study, we genotyped a panel of 133 soybean landraces using 82,187 SNPs and surveyed four yield-related traits at the R6 stage in two consecutive years. The objectives of this study were to (1) reveal the genetic basis of yield-related traits in soybean at the R6 stage and (2) provide valuable markers and candidate genes for the molecular breeding of vegetable soybean.

## Methods

### Plant materials and field trials

A total of 133 soybean landraces came from the soybean mini core collection, and the soybean mini core collection were selected from 23,587 soybean germplasms in the Chinese National Soybean GeneBank. Thus the 133 soybean landraces had abundant genetic diversity and were suitable for association analysis [24]. The 133 soybean germplasms came from 24 provinces and were distributed in four ecoregions of China as follows: The Northeast region (NER), the North region (NR), the Huanghuai region (HHR) and the South region (SR) (Additional file 1: Table S1).

These germplasms included abundant genetic diversity due to geographic, climatic and cultivation factors present in China and could be used for GWAS analysis. They were planted at the Jiangpu Experimental Station of the Agricultural University of Nanjing, China (32.04°N 118.63°E) in late June 2015 and 2016, according to a completely randomized block design, with two years and three replications. Planting was performed with two rows per plot and 40 plants per row, with plant spacing of 10 cm and row spacing of 50 cm.

### Phenotypic evaluation and statistical analysis

Four yield-related traits, the 100-pod fresh weight (PFW), 100-seed fresh weight (SFW), 100-seed dry weight (SDW) and moisture content of fresh seeds (MCFS), were investigated at the R6 growth stage during which the pods contain full-size green beans. At least fifty pods were harvested for each replication in each year. The pods were then weighed on the electronic scale, and PFW (g) was calculated. Next, the pod husks were stripped, and seed weight was measured to determine SFW (g). The seeds were then killed by heating at 110 °C for 30 min and dried at 65 °C to a constant weight to obtain the SDW (g). Finally, MCFS (%) was calculated using the following formula.

$$ \mathrm{MCFS}\left(\%\right)=\frac{\mathrm{SFW}\left(\mathrm{g}\right)-\mathrm{SDW}\left(\mathrm{g}\right)}{\mathrm{SFW}\left(\mathrm{g}\right)}\times 100\% $$

Statistical analyses for all traits were performed using SAS version 9.4 [25]. Analysis of variance (ANOVA) of the phenotypic data across multiple environments was performed using PROC GLM. The statistical model was as follows: *y*_*ijk*_ = *μ* + *α*_*i*_ + *β*_*j*_ + *γ*_*kj*_ + (*αβ*)_*ij*_ + *ε*_*ijk*_, where *μ* is the overall mean, *α*_*i*_ is the genetic effect of the i^th^ genotype, *β*_*j*_ is the effect of the j^th^ environment, *γ*_*kj*_ is the random effect of the k^th^ replicate in the j^th^ environment, *(αβ)*_*ij*_ is the interaction effect between the i^th^ genotype and the j^th^ environment, and *ε*_*ijk*_ is the residual. As sources of variation, the environment, genotype, replication within environment, and genotype × environment were treated as random effects. The formula for calculating broad-sense heritability is:

${h}^2={\alpha}_g^2/\left({\alpha}_g^2+{\alpha}_{ge}^2/n+{\alpha}_{\varepsilon}^2/ rn\right)$, *σ*^*2*^_*g*_ is the genotypic variance, *σ*^*2*^_*ge*_ is the genotype by environment interaction variance, *α*^*2*^_*ε*_ is the error variance, *n* is the number of environments, and *r* is the number of replications. All of the above variance values can be calculated using the REML method for the SAS VARCOMP procedure.

### SNP genotyping

The association panel was genotyped using a 180 K AXIOM® SoyaSNP array [26], and a total of 169,028 high-quality single nucleotide polymorphisms (SNPs) were used for association mapping. In this study, SNPs with minor allelic frequencies (MAFs) of less than 5% and a missing rate of more than 10% were excluded from further analysis. As a result, 82,187 SNPs remained and were used in marker-trait association analysis. The density of the SNPs was estimated as one SNP per 11.76 kb for the 20 soybean chromosomes.

### Population structure and linkage disequilibrium

We used PLINK V1.07 to perform SNP filtering by setting the MAF to 0.2 and the call rate to 0.1. The remaining data contained 8270 SNPs, which were used to construct a population structure in STRUCTURE 2.3.4. The number of subgroups (K) was set from 1 to 6, with 4 replications. The length of the burn-in period was set to 10,000, and the number of Monte Carlo Markov Chain (MCMC) replications was set to 100,000. The suitable K in this population was determined by the log probability of the data LnP(D) and delta K. In previous studies, the mini core collection was divided into two or three distinct subgroups depending on the markers used in the tests [24, 27, 28].

A total of 82,187 SNPs (MAF > 0.05) were employed to conduct principal component analysis (PCA) and construct a neighbor-joining (NJ) phylogenetic tree using PLINK V1.07 and PHYLIP. The kinship matrix was assessed using TASSEL V5.2.15 to determine the relatedness among individuals based on the SNP dataset [29]. Linkage disequilibrium parameters (*r*^*2*^) for estimating the degree of LD between pairwise SNPs (MAF > 0.2) were calculated using PLINK V1.07, and a figure showing average LD decay was drawn with R [30]. The LD decay rate of the population was measured as the chromosomal distance when the average *r*^*2*^ decreased to half its maximum value [31].

### Association mapping

The population structure and relative kinship in natural populations always result in a high level of spurious positives in association mapping [32]. After assessment of the population structure (Q), PCA, and evaluation of the relative kinship (K) of 133 soybean landraces, the effects of these parameters on association analyses were evaluated with the following statistical models: (1) a general linear model (GLM) with Q; (2) GLM with PCA; (3) a mixed linear model (MLM) with PCA and K; (4) and MLM with Q and K. Genome-wide association analyses were performed by TASSEL V5.2.15. The significance threshold for SNP-trait associations was determined by 1/n where n is the number of markers in the association panel, and *P* ≤ 1/82,187, or –Log_10_(*P*) ≥ 4.91 [33].

### Prediction of candidate genes

To identify candidate genes underlying the association signals, we selected significant SNPs associated with large-effect QTLs to search candidate genes in their candidate regions. The candidate regions were defined by the average LD decay distance or the LD block. The soybean reference genome was Wm82.a2.v1, and the functional annotations and tissue expression of genes located in the candidate regions were obtained from Phytozome (http://www.phytozome.net). Based on the soybean genomic annotations and expression data, potential candidate genes were predicted.

To determine the expression of potential candidate genes, we used quantitative real-time PCR (qRT-PCR) to analyze their expression in extreme materials with large phenotypic differences. Based on the phenotypic data in 2015 and 2016, the materials (ZDD21907 (PFW 198 g), ZDD20532 (PFW 39 g), ZDD01983 (MCFS 75.5%) and ZDD02315 (MCFS 61.7%)) showed stable and large phenotypic differences, therefore we chosen them as the extreme materials and cultivated in the field. Three replicate biological samples were collected in liquid nitrogen at three stages during soybean seed development (R5(3-mm-long seeds in a pod at one of the four uppermost nodes on the main stem, with a fully developed leaf), R6 (pods containing green seeds that fill the pod cavity, located at one of the four uppermost nodes on the main stem, with a fully developed leaf) and R7 (one normal pod on the main stem that has reached the mature pod color)), as defined by Fehr (1977) [34]. Total RNA was extracted from R5, R6, and R7 seeds using a RNA Simple Total RNA kit (TIANGEN, China). cDNA was synthesized using a Prime Script™ RT Reagent Kit (TaKaRa, Japan) with a standard protocol. The CDS sequences of the potential candidate genes were obtained from Phytozome (http://www.phytozome.net). The qRT-PCR primers were designed with Primer Premier 5.0 and were listed in Additional file 2: Table S2. *GmEF1β* (GenBank ID AK286947.1) was selected as the control gene, and the qRT-PCR assays were conducted three times using a Light Cycler 480 instrument. The relative expression level of the candidate genes was calculated using the comparative 2^−△△CT^ method [35]. Statistical analyses were performed with Dunnett’s tests and Student’s t-tests.

## Results

### Phenotypic analysis of four yield-related traits

**Fig. 1:**
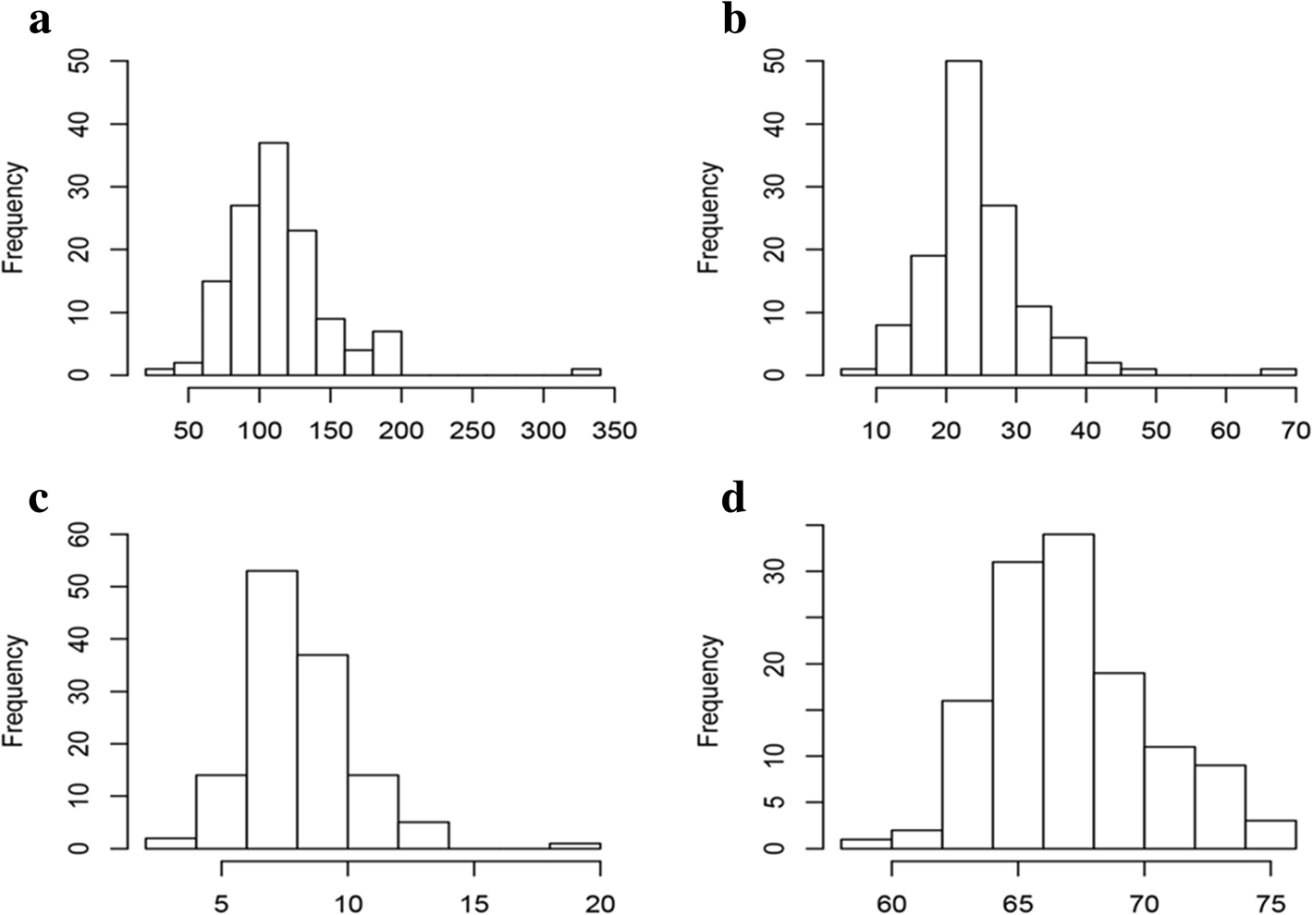
Distribution of four yield-related traits, showing mean values across 2 years: **a** 100-pod fresh weight (g), **b** 100-seed fresh weight, **c** 100-seed dry weight, and **d** moisture content of fresh seeds

A total of 133 soybean landraces were planted in two consecutive years, and four yield-related traits were investigated. The average values of these traits across the two years showed a continuous distribution in the GWAS panel of 133 soybean landraces, with a wide range of variation (Table 1). PFW exhibited 9.25-fold variation, ranging from 35.9 g to 332.1 g, with an average of 118.2 ± 39.2 g. SFW and SDW showed approximately 8-fold differences, ranging from 8.7 g to 72.4 g and 2.7 g to 21.7 g, respectively. MCFS showed 1.38-fold variation, ranging from 57.0 to 79.0%, with an average of 66.0 ± 4.0%. The frequency distribution of the four yield-related traits displayed an approximately normal distribution except for a few materials that showed large deviation (Fig. 1). According to the method described by Wyman (1991) [36], the broad-sense heritability (*h*^*2*^) was calculated for the four traits. All traits presented an *h*^*2*^ above 82%, suggesting that genetic effects play a predominant role in the phenotype variation of these traits (Table 1). Phenotypic correlations were analyzed between the four traits, and most exhibited significant positive correlations with each other (*p* < 0.05; Table 2). Highly significant positive correlations were observed between PFW, SFW and SDW, with phenotypic correlation coefficients (*r*_*p*_) above 0.914. MCFS showed a significant positive correlation with PFW and SFW (*r*_*p*_ = 0.205, *r*_*p*_ = 0.245) but showed a nonsignificant negative correlation with SDW, suggesting that MCFS is an important factor influencing the yield of fresh pods.

**Table 1:** Statistics of 100-pod fresh weight (PFW), 100-seed fresh weight (SFW), 100-seed dry weight (SDW) and moisture content of fresh seeds (MCFS) for the 133 soybean landraces

Traits	Mean ± SD	Range	F^a^_G_	F^a^_E_	F^a^_GxE_	Heritability^b^(%)
PFW(g)	118.2 ± 39.2	35.9–332.1	84.7^***^	98.6^***^	2.9^***^	96.7
SFW(g)	27.3 ± 8.6	8.7–72.4	68.0^***^	1121.8^***^	4.5^***^	93.5
SDW(g)	9.2 ± 2.7	2.7–21.7	30.9^***^	985.8^***^	3.9^***^	87.6
MCFS(%)	66.0 ± 4.0	57.0–79.0	15.0^***^	197.9^***^	2.6^***^	83.1

^a^F_G_, F_E_, and F_GxE_ represent the F value for genotypic, environmental effects and genotype × environment interaction, respectively

^b^Entry mean-based heritability: *H*^*2*^ = σ^2^_g_/[σ^2^_g_ + σ^2^_ge_/n + σ^2^_ε_/(rn)], where σ^2^_g_ is the genotypic variance, σ^2^_ge_ is the genotype by environment interaction variance, σ^2^_ε_ is the error variance, n is the number of environments, r is the number of replications

*** Significant at *p* <0.001

**Table 2:** Correlation coefficients among four yield-related traits

Traits	PFW	SFW	SDW	MCFS
PFW	1			
SFW	0.962^**^	1		
SDW	0.914^**^	0.939^**^	1	
MCFS	0.205^*^	0.245^**^	−0.085	1

The average across two years was used to calculate the correlation coefficients. PFW (100-pod fresh weight), SFW (100-seed fresh weight), SDW (100-seed dry weight), MCFS (Moisture content of fresh seeds). * Significant at *P* < 0.05, ** Significant at *P* < 0.01

### Distribution of markers and linkage disequilibrium

**Fig. 2:**
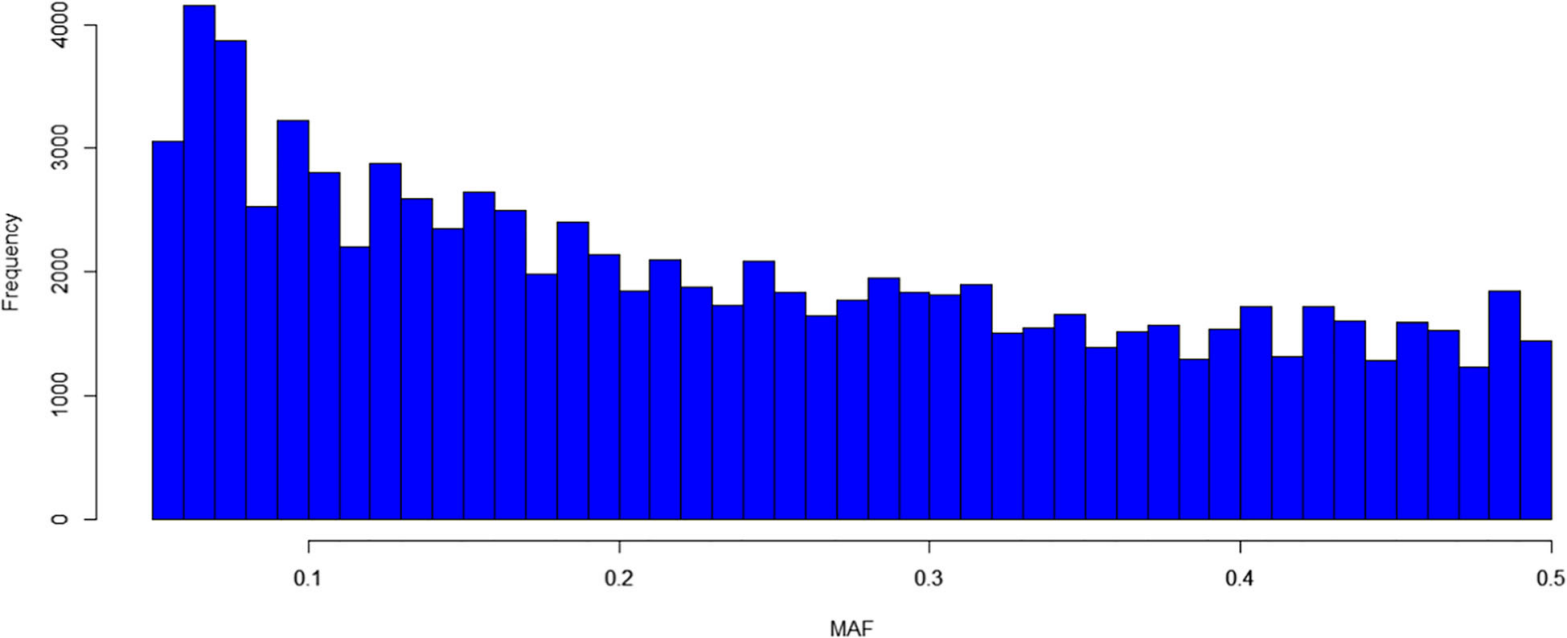
Minor allelic frequency distribution in 133 soybean landraces based on 82,187 SNPs

A total of 82,187 high-quality SNPs (MAF > 0.05, missing rate < 10%) were used for a GWAS of the four traits, with an average marker density of 11.76 kb/SNP at the genome-wide scale. The lowest marker density (16.28 kb/SNP) was found on Chr.14, and the highest marker density (9.57 kb/SNP) was found on Chr.16. Thus, the markers were unevenly distributed throughout the genome (Additional file 3: Table S3). The MAFs of the 82,187 SNPs are shown in Fig. 2. The average MAF was 0.24, and most of the SNPs (60.5%) exhibited an MAF higher than 0.2. The mean gene diversity (GD) was 0.37, and the values ranged from 0.34 to 0.40. The polymorphism information content (PIC) of all markers ranged from 0.29 to 0.33, with an average of 0.31 (Additional file 3: Table S3).

**Fig. 3:**
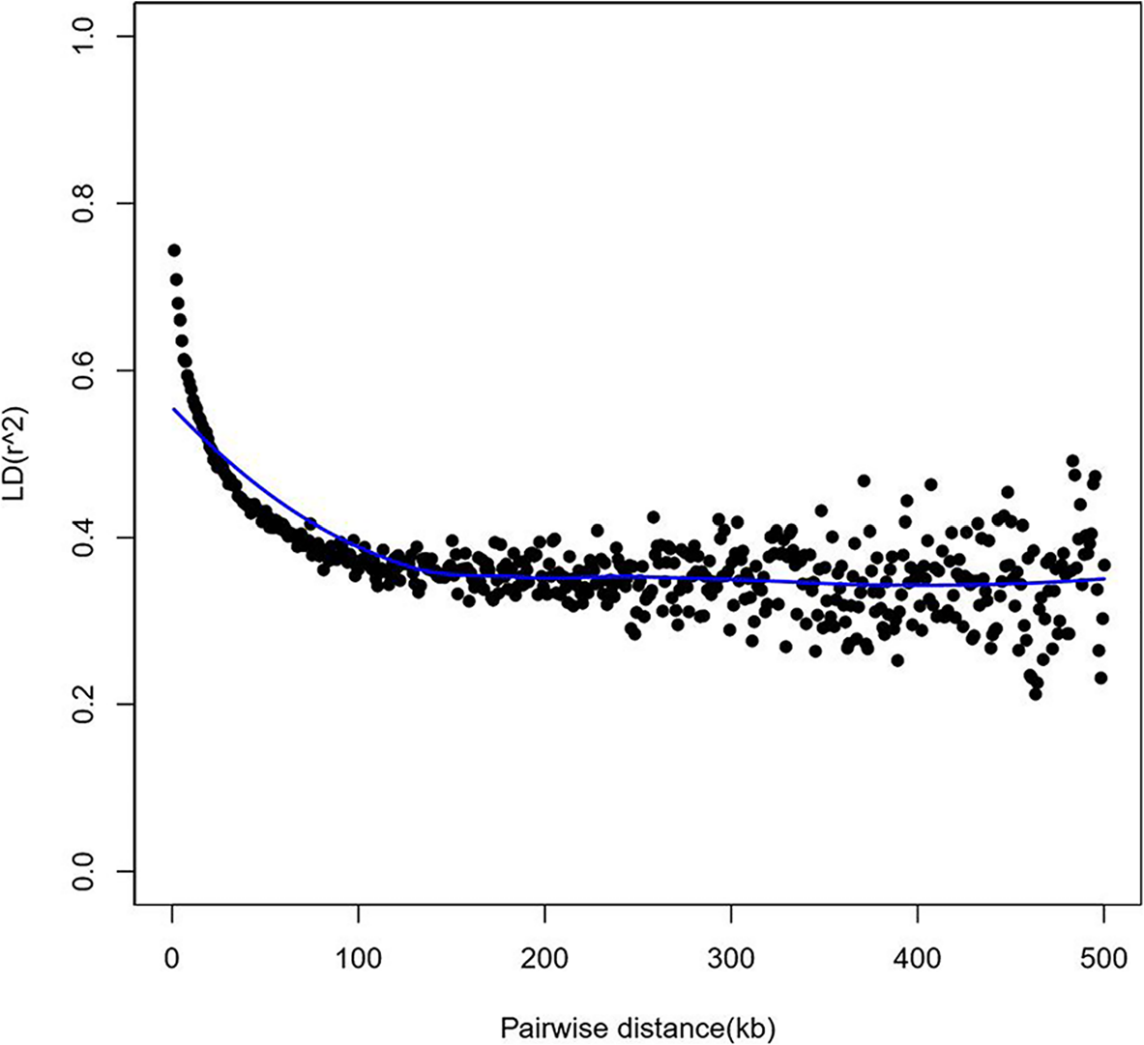
Average linkage disequilibrium (LD) decay rate estimated among co-chromosome SNPs

Genome-wide LD decay in the association panel was estimated. A rapid decline in LD was observed with increasing physical distance between pairwise SNPs. The mean length of LD decay decreased rapidly to 21 kb at a cut-off of *r*^*2*^ = 0.5. The overall LD decay for all chromosomes was estimated as 119.07 kb, where *r*^*2*^ = 0.375 (half of its maximum value) (Fig. 3).

### Population structure analysis

**Fig. 4:**
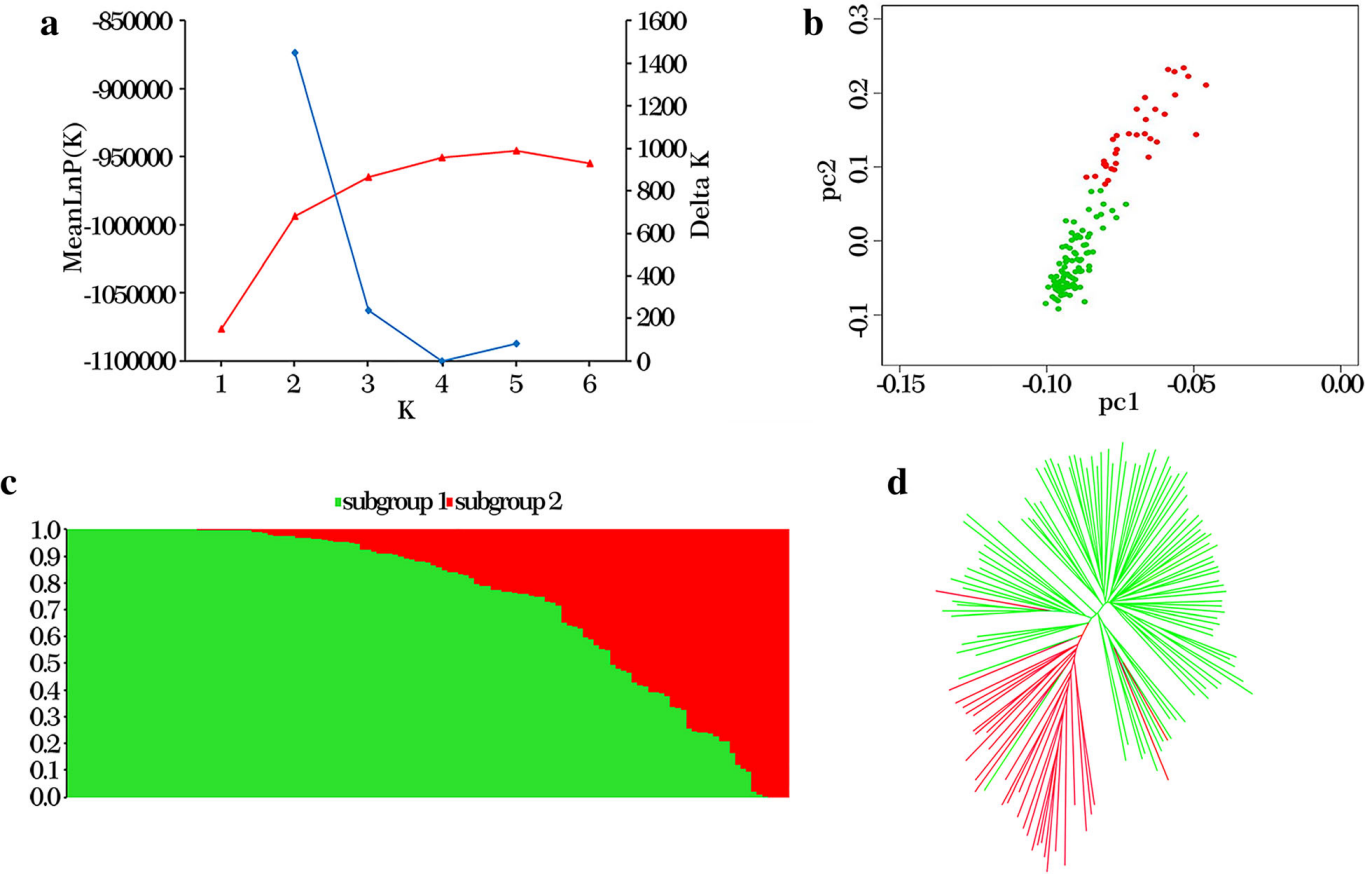
Population structure analysis of 133 soybean landraces. **a** The mean LnP(k) and Delta k values when k ranges from 1 to 6. **b** Two-dimensional scatter plot of PCA, the green dot represents subgroup 1 and the red dot represents subgroup 2. **c** Population structure of 133 soybean landraces, there are two colored segments and each segment represents the percentage of the individual in the population. **d** A neighbor-joining tree of the 133 soybean landraces that can be divided into two subgroups

Population structure analysis showed that the mean LnP (K) did not plateau at a single k value but instead continued to increase with relatively constant increments. Calculation of Delta K revealed a sharp peak at k = 2; therefore, the 133 soybean landraces were divided into two subgroups, designated subgroup1 and subgroup2 (Fig. 4a and c). The geographical origins of the 133 soybean landraces were the Northeast region (NER), the North region (NR), the Huanghuai region (HHR) and the South region (SR). Subgroup 1 contained 101 accessions; among these, 63 accessions belonged to SR, 21 accessions belonged to HHR, 5 accessions belonged to NR, and 12 accessions belonged to NER. Subgroup 2 was small and included only 32 accessions; among these, 2 accessions belonged to SR, 10 accessions belonged to HHR, 13 accessions belonged to NR, and 7 accessions belonged to NER (Additional file 4: Table S4). Notably, most accessions from SR (97%) were included in subgroup 1, whereas most accessions from NR (72%) were included in subgroup 2, suggesting that the population stratification of the 133 accessions essentially corresponded to their geographic origins. The NJ tree and PCA provided further support for the population structure results (Fig. 4b and d).

### Model comparison for the control of false associations

**Fig. 5:**
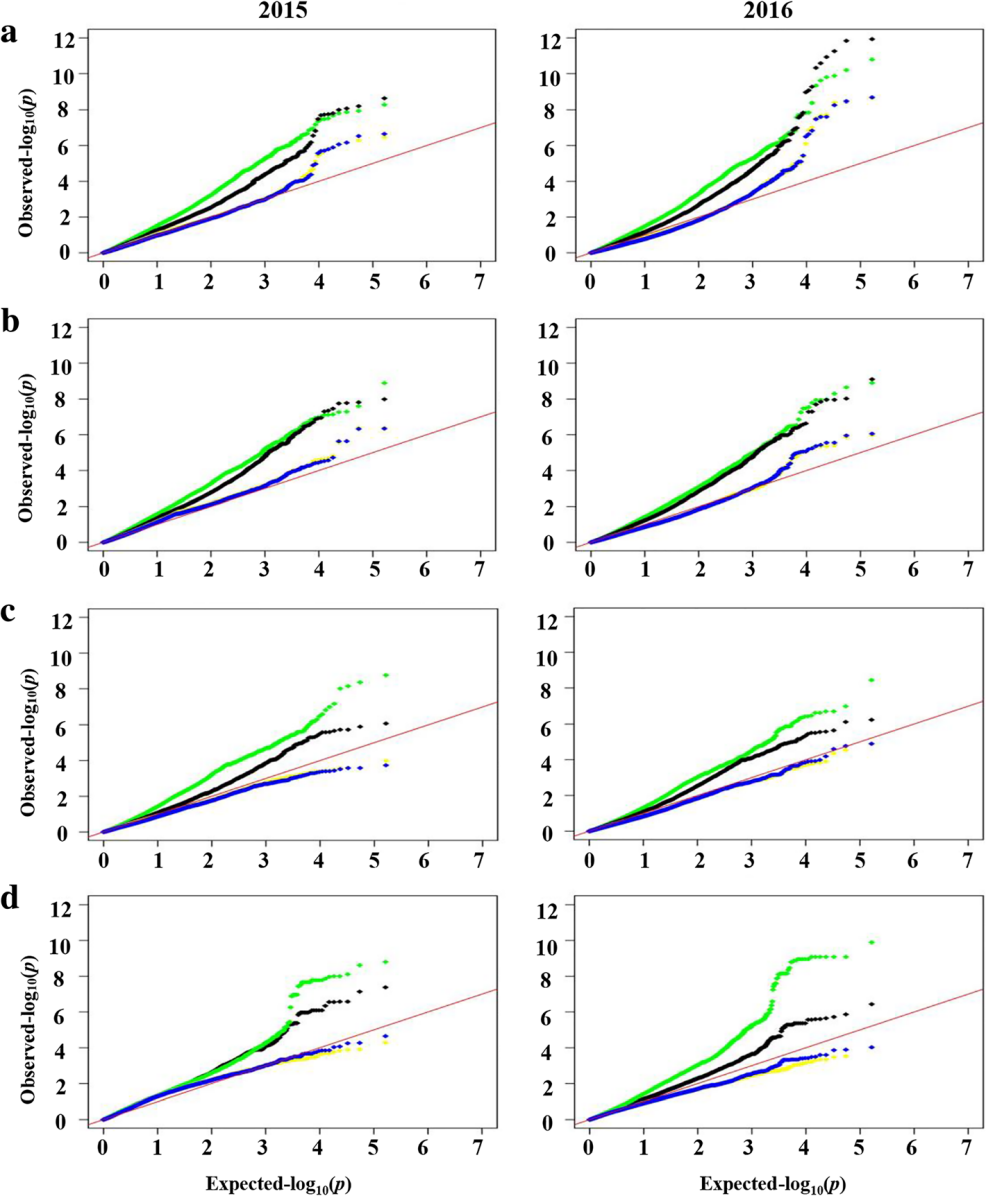
Quantile–quantile plots of estimated −log_10_ (*P*) from association analysis of four yield-related traits in two years (2015 and 2016): **a** 100-pod fresh weight, **b** 100-seed fresh weight, **c** 100-seed dry weight, and **d** moisture content of fresh seeds. Red line represents expected *P* values with no association. The black line represents the observed *P* values using the GLM (PCA) model. The green line represents the observed *P* values using the GLM (Q) model. The yellow line represents the observed *P* values using the MLM (PCA + K) model. The blue line represents the observed *P* values using the MLM (Q + K) model

Association analyses for the four yield-related traits were performed to evaluate the effects of different models on the control of false associations. For PFW and SFW, the observed *P* values from the GLM(Q) model showed the greatest deviation from the expected *P* values assuming that no association exists, followed by the GLM (PCA) model. The *P* values from the MLM (Q + K) and MLM (PCA + K) models were similar and close to the expected *P* values, and the effects of the MLM (Q + K) and MLM (PCA + K) models on the controlling false associations were similar (Fig. 5). For SDW and MCFS, the observed *P* values from the MLM (PCA + K) and MLM (Q + K) models were lower than the expected *P* values, suggesting that the two models excessively corrected the observed *P* values; thus, no significant associations were identified. The observed *P* values from the GLM (PCA) and GLM (Q) models were higher than the expected P values, and the observed *P* values from GLM (PCA) were much closer to the expected *P* values than those from the GLM (Q) model, indicating that the GLM (PCA) model could effectively control false-positive associations and avoid false-negative associations. Thus, for PFW and SFW, the MLM (Q + K) model was chosen for subsequent association analyses, whereas for SDW and MCFS, the GLM (PCA) model was selected.

### Genome wide association analysis of four yield-related traits

Using GWAS, a total of 111 and 146 associations (−Log_10_(*P*) > 4.91) were evaluated for the four yield-related traits using the means across 2 years and within individual years, respectively (Additional file 5: Table S5). The resultant quantile–quantile plots and Manhattan plots are shown in Additional file 6: Figure S1, Additional file 7: Figure S2, Additional file 8: Figure S3 and Additional file 9: Figure S4. For PFW, fourteen SNPs were detected (Additional file 5: Table S5). Among these SNPs, nine were repeatedly detected in all environments and were distributed on 7 of 20 soybean chromosomes, and the contribution of a single marker to the observed phenotypic variation was 25.12–33.61% (Table 3). AX-90496773 presented the largest phenotypic difference of 16.33 g between alleles, with an effect on PFW (*R*^*2*^ = 29.99%). For SFW, fifteen significant SNPs were detected (Additional file 5: Table S5). Among these SNPs, only four were repeatedly detected in all environments, and each SNP could explain a large proportion (26.54–27.8%) of the phenotypic variance (Table 3). AX-90519309 had a large effect (*R*^*2*^ = 27.47%) on SFW, with variance of 1.74 g between alleles. Sixty-three SNPs were significantly associated with SDW (Additional file 5: Table S5). Among these, eight SNPs were repeatedly detected in all environments (Table 3). AX-90501040 had the largest effect (*R*^*2*^ = 24.87%) on SDW, associated with a difference of 5.81 g between alleles. For MCFS, a total of forty-eight SNPs were identified (Additional file 5: Table S5). Of these, twenty SNPs were repeatedly detected in all environments, and all were located in a range of 164 kb (41791118–41,955,229) on chromosome 5 (Table 3). AX-90435701 and AX-90460290 had the largest effect (*R*^*2*^ = 21.56%) on MCFS, associated with a difference of 3.51% between alleles. Altogether, thirty-five markers were repeatedly associated with one of the four yield-related traits in all environments. In addition, four markers (AX-90490395, AX-90481424, AX-90370125 and AX-90519309) were commonly associated with both PFW and SFW, and two markers (AX-90328574 and AX-90496773) were associated with both PFW and SDW in all environments (Table 3). However, no markers overlapped between MCFS and the other three traits.

**Table 3:** SNPs signifcantly associated with the four yield-related traits and previously reported QTLs at similar genome regions

Traits	SNP^a^	MAF^b^	Physical position		Significant region		Mean^c^		Effect^d^	Env^e^	Known QTLs^f^
			Chr.	Position	Start	End	-Log_10_(*P*)	*R*^*2*^(%)			
PFW	AX-90490395	0.34	2	46,440,530	46,321,460	46,559,600	6.41	27.72	3.81	15,16,mean	seed weight 50–12
	AX-90483564	0.27	3	36,787,728	36,668,658	36,906,798	6.60	29.19	4.57	15,16,mean	
	AX-90435834	0.18	4	1,402,717	1,283,647	1,521,787	6.08	25.12	11.06	15,16,mean	Seed weight per plant 6–2; Seed weight 47–3; Seed height 1–12, Seed length 1–13
	AX-90328574	0.11	9	39,625,218	39,506,148	39,744,288	6.52	27.10	11.46	15,16,mean	Seed weight 50–5; Seed yield 31–10
	AX-90481424	0.24	14	5,733,475	5,614,405	5,852,545	6.52	27.22	4.58	15,16,mean	Seed weight 36–14
	AX-90512978	0.14	14	45,661,649	45,542,579	45,780,719	6.49	27.16	15.43	15,16,mean	Seed yield 31–1
	AX-90496773	0.07	16	1,617,227	1,498,157	1,736,297	7.03	29.99	16.33	15,16,mean	Seed yield 23–6; Pod maturity 19–6; Pod maturity 9–1
	AX-90370125	0.08	16	5,791,933	5,672,863	5,911,003	7.54	33.61	0.53	15,16,mean	Seed yield 29–2
	AX-90519309	0.35	17	4,197,693	4,078,623	4,316,763	7.34	32.14	8.22	15,16,mean	Seed weight 21–2; Seed weight 22–3; Seed weight 22–4
SFW	AX-90490395	0.34	2	46,440,530	46,321,460	46,559,600	6.26	26.86	0.64	15,16,mean	Seed weight 50–12
	AX-90481424	0.24	14	5,733,475	5,614,405	5,852,545	6.45	26.54	0.75	15,16,mean	Seed weight 36–14
	AX-90370125	0.08	16	5,791,933	5,672,863	5,911,003	6.37	27.80	0.01	15,16,mean	Seed yield 29–2
	AX-90519309	0.35	17	4,197,693	4,078,623	4,316,763	6.35	27.47	1.74	15,16,mean	Seed weight 21–2;, Seed weight 22–3, Seed weight 22–4
SDW	AX-90505318	0.12	1	50,248,686	50,129,616	50,367,756	7.31	17.69	3.71	15,16,mean	Seed weight 15–2; Seed weight 45–2
	AX-90395822	0.14	1	50,267,101	50,148,031	50,386,171	7.12	19.42	3.79	15,16,mean	Seed weight 15–2; Seed weight 45–2
	AX-90328574	0.11	9	39,625,218	39,506,148	39,744,288	5.84	16.24	0.48	15,16,mean	Seed weight 50–5; Seed yield 31–10
	AX-90501040	0.05	14	42,496,533	42,377,463	42,615,603	9.58	24.87	5.81	15,16,mean	Seed yield 32–3; Pod maturity 27–3
	AX-90367415	0.05	14	42,696,630	42,577,560	42,815,700	6.58	18.05	3.69	15,16,mean	Seed yield 32–3; Pod maturity 27–3
	AX-90480993	0.05	14	42,700,090	42,581,020	42,819,160	6.58	18.05	3.69	15,16,mean	Seed yield 32–3; Pod maturity 27–3
	AX-90496773	0.07	16	1,617,227	1,498,157	1,736,297	6.16	17.29	0.60	15,16,mean	Seed yield 23–6; Pod maturity 19–6; Pod maturity 9–1
	AX-90421382	0.09	16	5,520,943	5,401,873	5,640,013	5.51	13.46	2.76	15,16,mean	Seed yield29–2
MCFS	AX-90441957	0.46	5	41,791,118	41,672,048	41,910,188	7.38	19.52	3.39	15,16,mean	Seed thickness 1–3; Seed arabinose plus galactose 1–1; Seed yield 15–3
	AX-90371675	0.47	5	41,797,554	41,678,484	41,916,624	7.36	19.32	3.39	15,16,mean	Seed thickness 1–3; Seed arabinose plus galactose 1–1; Seed yield 15–3
	AX-90525251	0.46	5	41,807,238	41,688,168	41,926,308	7.38	19.52	3.39	15,16,mean	Seed thickness 1–3; Seed arabinose plus galactose 1–1; Seed yield 15–3
	AX-90320946	0.47	5	41,807,727	41,688,657	41,926,797	7.36	19.32	3.39	15,16,mean	Seed thickness 1–3; Seed arabinose plus galactose 1–1; Seed yield 15–3
	AX-90347760	0.46	5	41,808,982	41,689,912	41,928,052	7.38	19.52	3.39	15,16,mean	Seed thickness 1–3; Seed arabinose plus galactose 1–1; Seed yield 15–3
	AX-90335134	0.46	5	41,815,650	41,696,580	41,934,720	7.38	19.52	3.39	15,16,mean	Seed thickness 1–3; Seed arabinose plus galactose 1–1; Seed yield 15–3
	AX-90418462	0.47	5	41,818,004	41,698,934	41,937,074	7.36	19.32	3.39	15,16,mean	Seed thickness 1–3; Seed arabinose plus galactose 1–1; Seed yield 15–3
	AX-90492796	0.47	5	41,818,197	41,699,127	41,937,267	7.36	19.32	3.39	15,16,mean	Seed thickness 1–3; Seed arabinose plus galactose 1–1; Seed yield 15–3
	AX-90363684	0.47	5	41,818,450	41,699,380	41,937,520	7.36	19.32	3.39	15,16,mean	Seed thickness 1–3; Seed arabinose plus galactose 1–1; Seed yield 15–3
	AX-90392895	0.46	5	41,828,027	41,708,957	41,947,097	7.38	19.52	3.39	15,16,mean	Seed thickness 1–3; Seed arabinose plus galactose 1–1; Seed yield 15–3
	AX-90333199	0.46	5	41,831,486	41,712,416	41,950,556	7.18	18.99	3.36	15,16,mean	Seed thickness 1–3; Seed arabinose plus galactose 1–1; Seed yield 15–3
	AX-90344127	0.44	5	41,833,722	41,714,652	41,952,792	7.64	20.59	3.36	15,16,mean	Seed thickness 1–3; Seed arabinose plus galactose 1–1; Seed yield 15–3
	AX-90494650	0.46	5	41,853,747	41,734,677	41,972,817	7.36	19.32	3.36	15,16,mean	Seed thickness 1–3; Seed arabinose plus galactose 1–1; Seed yield 15–3
	AX-90424180	0.47	5	41,866,507	41,747,437	41,985,577	7.75	20.30	3.47	15,16,mean	Seed thickness 1–3; Seed arabinose plus galactose 1–1; Seed yield 15–3
	AX-90335974	0.46	5	41,882,999	41,763,929	42,002,069	7.00	20.68	3.51	15,16,mean	Seed thickness 1–3; Seed arabinose plus galactose 1–1; Seed yield 15–3
	AX-90435701	0.47	5	41,903,235	41,784,165	42,022,305	7.34	21.56	3.51	15,16,mean	Seed thickness 1–3; Seed arabinose plus galactose 1–1; Seed yield 15–3
	AX-90460290	0.47	5	41,927,984	41,808,914	42,047,054	8.19	21.56	3.51	15,16,mean	Seed thickness 1–3; Seed arabinose plus galactose 1–1; Seed yield 15–3
	AX-90337409	0.47	5	41,932,683	41,813,613	42,051,753	8.09	21.17	3.47	15,16,mean	Seed thickness 1–3; Seed arabinose plus galactose 1–1; Seed yield 15–3
	AX-90391337	0.48	5	41,947,344	41,828,274	42,066,414	7.36	19.34	3.37	15,16,mean	Seed thickness 1–3; Seed arabinose plus galactose 1–1; Seed yield 15–3
	AX-90415951	0.48	5	41,955,229	41,836,159	42,074,299	7.36	19.34	3.37	15,16,mean	Seed thickness 1–3; Seed arabinose plus galactose 1–1; Seed yield 15–3

^a^The significant SNP ID, ^b^Minor allele frequency for each associated marker, ^c^The average across two years was used to association analysis -Log_10_(*P*) and *R*^*2*^ were listed, ^d^Phenotypic differences between different genotypes classified on alleles of associated markers, ^e^15 and 16 represented the environments of years 2015 and 2016, respectively. “mean” represented associations detected with the mean values across two years, ^f^Comparision of trait-marker associations identified in this study with QTLs identified in previous studies. Based on the QTL list on SoyBase (http://www.soybase.org), The underlined SNPs were common markers detected in two traits

### Prediction of candidate genes

**Fig. 6:**
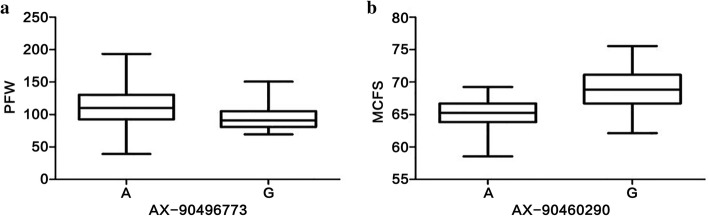
Phenotypic differences between accessions carrying different alleles. **a** The allele effects for the PFW marker AX-90496773 in soybean accessions. **b** The allele effects for MCFS  marker AX-90460290 in soybean accessions. PFW means 100- pod fresh weight, MCFS means moisture content of fresh seeds

**Fig. 7:**
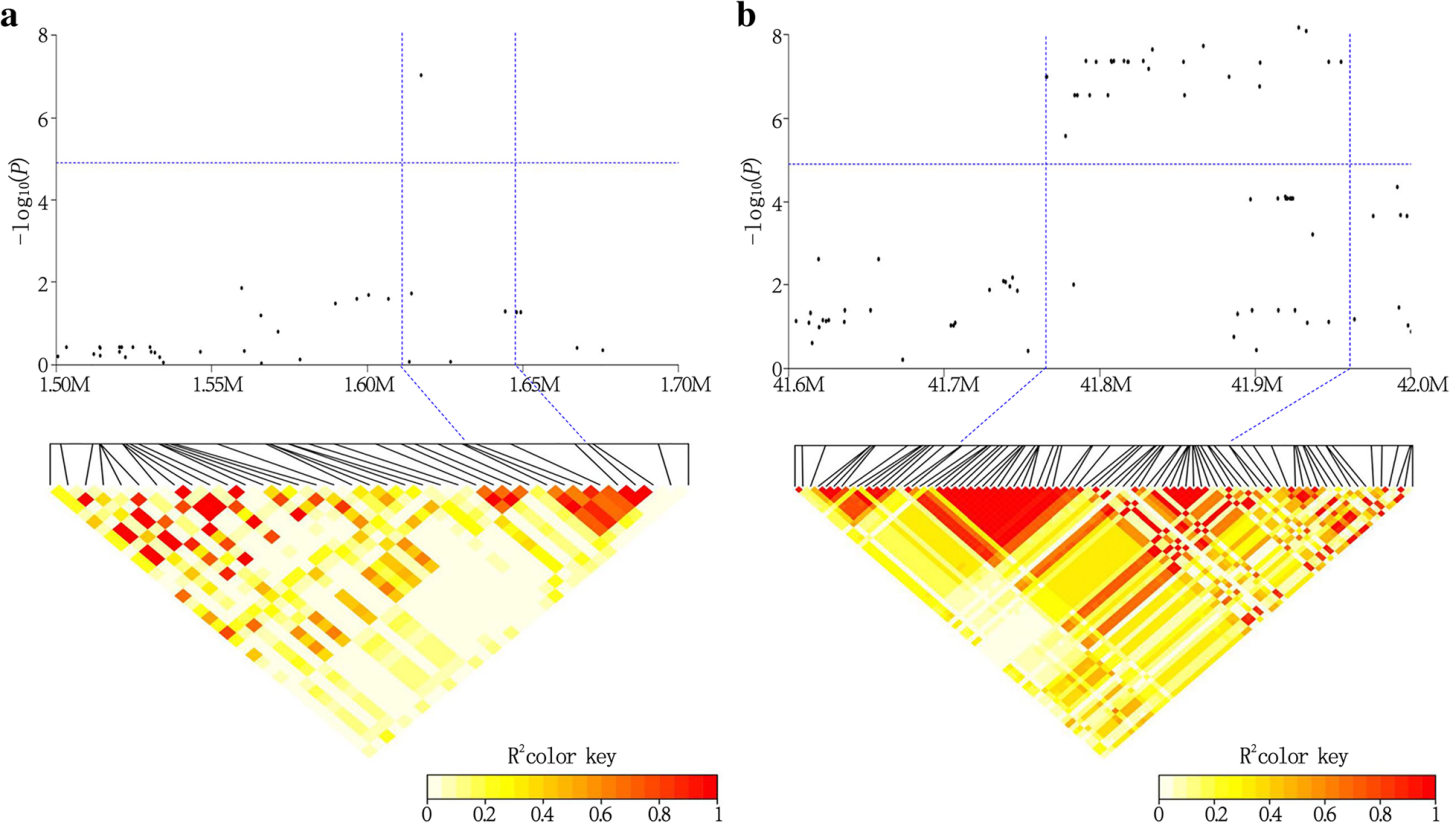
The candidate regions of the large-effect markers which associated with the 100-pod fresh weight (PFW) and moisture content of fresh seeds (MCFS) in soybean. **a** AX-90496773 which associated with PFW is located on Gm16. **b** AX-90460290 which associated with MCFS is located on Gm19. In the top panel, negative log_10_-transformed *P* values of SNPs from GWAS for PFW and MCFS are plotted against the physical positions of the given chromosomal regions. The bottom panel depicts the extent of LD in this region based on *r*^*2*^, and the color key displays *r*^*2*^ values. The horizontal dashed line (in blue) indicates the significant threshold of the genome wide association analysis (-log_10_(*p*) >4.91). The candidate region for the locus is indicated by two vertical dashed blue lines

**Fig. 8:**
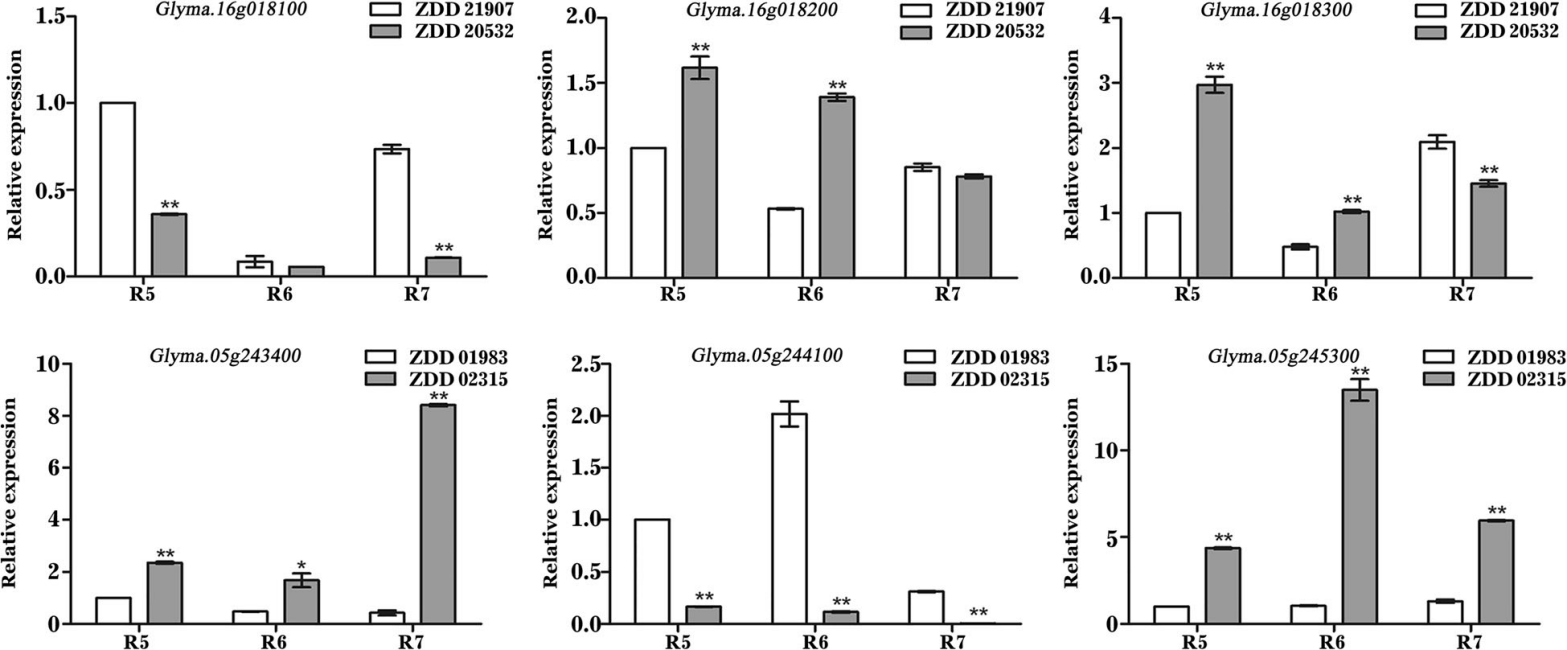
Expression analysis of potential candidate genes in extreme materials at three growth developmental stages (R5, R6 and R7). The extreme materials include ZDD21907 (PFW 198 g), ZDD20532 (PFW 39 g), ZDD01983 (MCFS 75.5%) and ZDD02315 (MCFS 61.7%). The error bar indicates standard deviation. The results are representative of three biological replicates. ∗ Significant at *P* < 0.05; ∗∗ Significant at *P* < 0.01

In this study, we were particularly interested in the markers with large effects, such as the PFW marker AX-90496773 (Gm16_1,617,227, MAF = 0.07) on chromosome 16, and the MCFS marker AX-90460290 (Gm05_41,927,984, MAF = 0.47) on chromosome 5. Compared with the alternative alleles, the PFW of the materials carrying the favorable allele (AA) at AX-90496773 was 16.33g higher than the materials carrying the unfavorable allele (GG), the MCFS of the materials carrying the favorable allele (GG) at AX-90460290 was 3.51% higher than the materials carrying the unfavorable allele (AA) (Fig. 6). LD analysis showed that AX-90496773 and AX-90460290 can be mapped to chromosomal regions of 34.5 kb on Gm16 and 189.1 kb on Gm05, respectively (Fig. 7). Within the regions of AX-90496773 and AX-90460290, there were five and twenty-seven putative genes, respectively. According to the functional annotations and the expression patterns of these putative genes from the Phytozome website (http://www.phytozome.net), we were able to initially predict potential candidate genes for PFW and MCFS. A total of six genes were considered potential candidate genes, and the functional annotations of these genes are listed in Table 4. To confirm the potential candidate genes whether participated in the accumulation of PFW or MCFS, we tested the expression patterns of the six genes via RT-qPCR in the seeds of extreme materials at three developmental growth stages (R5, R6 and R7). The genotype of extreme materials ZDD21907 (PFW 198 g) and ZDD20532 (PFW 39 g) at the AX-90496773 locus were AA (favourable allele) and GG (unfavourable allele), respectively. The genotype of extreme materials ZDD01983 (MCFS 75.5%) and ZDD02315 (MCFS 61.7%) at the AX-90460290 locus were GG (favourable allele) and AA (unfavourable allele), respectively. Among the three potential candidate genes associated with PFW, *Glyma.16g018200* and *Glyma.16g018300* showed significant differences in expression between ZDD21907 (PFW 198 g) and ZDD20532 (PFW 39 g) at the R5 and R6 stages (*P* ≤ 0.01) (Fig. 8). The potential candidate genes for MCFS were *Glyma.05g243400*, *Glyma.05g244100* and *Glyma.05g245300*. These three genes showed significant differences in expression between ZDD01983 (MCFS 75.5%) and ZDD02315 (MCFS 61.7%) at the R5, R6 and R7 stages (*P* ≤ 0.05, *P* ≤ 0.01) (Fig. 8). The differential expression of these genes in extreme materials provided support for the identification of candidate genes. Therefore, we speculate that *Glyma.16g018200* and *Glyma.16g018300* may be the candidate genes for PFW and that *Glyma.05g243400*, *Glyma.05g244100* and *Glyma.05g245300* may be the candidate genes for MCFS. To analyze the genetic mechanism of yield in vegetable soybean, we still need to further study these five genes.

**Table 4:** the function annotation and the high expression tissue of the potential candidate genes

Traits	Gene ID	Position (bp)	Annotation	High expression tissue^a^
PFW	*Glyma.16g018100*	1,612,068..1614560	Surfeit locus protein 2	pod
	*Glyma.16g018200*	1,617,162..1618781	Unknown	shoot apical meristem
	*Glyma.16g018300*	1,619,151..1623559	pyruvate dehydrogenase E1 component alpha subunit	seed
FGMC	Glyma.05g243400	41,800,695..41809429	Translation factor	seed
	Glyma.05g244100	41,852,863..41854961	phosphatidylethanolamine-binding proteins	seed
	Glyma.05g245300	41,925,667..41935273	Serine-threonine protein kinase	leave

^a^The tissue in which the gene had the highest expression level

## Discussion

Vegetable soybean, or edamame, is a specialty soybean harvested at the R6-R7 stage when pods are green and seeds are immature [37]. The seeds of vegetable soybeans are larger, sweeter and tender than those of grain soybeans, and because of their rich protein (33–39%) and low fat (13–16%) contents, they are increasingly popular among young people who seek healthy diets, especially in developed countries [38]. In addition, vegetable soybean is a good source of soluble sugar, dietary fiber, vitamin C, vitamin E, calcium, and phytoestrogens [39, 40]. With the social and economic development, there is a growing global demand for vegetable soybeans. Since the 1990s, the demand for vegetable soybeans has grown in the US, reaching 10,000 tons in 2000 [41]. Japan is the largest importer of vegetable soybean, with a total demand of more than 176,000 tons annually [42] However, the demand for vegetable soybean cannot be met due to a lack of excellent varieties. China is the country of origin for soybeans and possesses the most soybean genetic resources worldwide. Based on the abundance of soybean resources, GWAS have been conducted to dissect the genetic architecture of vegetable soybean yield, providing functional markers, beneficial genes and specific materials for the molecular design and breeding of vegetable soybeans.

The acceptable distance between the markers and the candidate genes was determined based on LD, which varies with species and populations [43]. In this study, the overall LD decay distance for the 133 soybean landraces was 119.07 kb (*r*^*2*^ = 0.375) across the entire genome, which was within the reported range (90 kb ~ 574 kb), but slightly lower than the previously reported distance of 130 kb in cultivated soybean [44]. Greater diversity of geographic origins (NR, HR, SR, and NER) was included in our GWAS panel, and this difference in geographic origin may be responsible for the relatively low LD found in this study. A low LD decay rate was also identified in another recent GWAS of soybeans, involving widely distributed geographic origins (China, Korea, Japan) [45]. Moreover, the 975 Mb soybean genome includes 54,175 putative genes annotated in the cultivated soybean genome [44]. On average, every 18.42 kb contains a gene, and the average SNP spacing was approximately 11.76 kb in our study (Additional file 3: Table S3); thus, it was theoretically sufficient for efficient GWAS analysis.

In previous studies, a total of 294 QTLs for seed weight were reported across the 20 soybean chromosomes (http://www.soybase.org/). In addition, many QTLs have been identified for several traits that are highly related to yield, such as seed size, flowering time, maturity and plant height. These QTLs could be used to confirm the loci identified by GWAS. In this study, the genetic bases of four yield-related traits at the R6 stage were analyzed using association mapping, and a total of 116 significant SNPs were identified (Additional file 5: Table S5). Of these SNPs, 35 were repeatedly detected in all environments (Table 3). The data indicated that a large majority of the SNPs were environment specific, and phenotypic plasticity plays an important role in plant agronomic diversity [46]. Each SNP associated with the yield at the R6 stage could explain a large proportion (> 13.46%) of the observed phenotypic variance (Table 3). This finding differs from the reported low phenotypic variance (< 4%) of each locus associated with seed weight at maturity [47]. The results demonstrated that the soybean yield at the R6 stage is a typical quantitative trait that is genetically conditioned by many large-effect loci. Thirty-four of the repeatedly identified SNPs have been shown to colocalize with QTLs identified in previously studies (Table 3). Among these SNPs, AX-90496773 at the 1.62 Mb position on Gm16 (a region similar to a previously reported seed yield 23–6 and pod maturity 9–1 and 19–6 QTLs) was strongly associated with both PFW and SDW. Another SNP, AX-90435834 at the 1.4 Mb position on Gm04, has been reported to colocalize with QTLs related to seed weight and seed size (e.g., seed weight per plant 6–2, seed weight 47–3, seed length 1–13 and seed height 1–12). The SNP AX-90519309 on Gm17, associated with PFW and SFW, was mapped within an overlapping region of three seed weight QTLs, indicating that AX-90519309 might be located in the hottest region related to soybean yield. Twenty SNPs associated with MCFS were mapped to a small region on Gm05. Three QTLs were previously reported in a similar region with seed yield 15–3, seed thickness 1–3 and Ara/Gal 1–1. Ara/Gal represents the ratio of arabinose and galactose contents and is significantly and negatively correlated with the average concentration of pectin [48]. Pectin is multifunctional, including functions in cell wall deposition and assembly, cell expansion, cell wall swelling and softening during fruit development [49]. Therefore, the region containing twenty significant SNPs might have an effect on seed moisture content and seed thickness by affecting seed pectin. The seed moisture content and seed thickness may influence soybean yield at the R6 stage. Fine mapping of such co-localized chromosomal regions would help to determine the candidate genes responsible for the natural variation of these yield-related traits.

In this study, a total of five candidate genes associated with PFW and MCFS at the R6 stage were predicted within the LD blocks of two markers of large effect (Fig. 7 and Table 4). Among these 5 genes, *Glyma.16g018200* and *Glyma.16g018300* are proposed as the candidate genes for PFW. The large-effect marker AX-90496773 is located in the CDS region of *Glyma.16g018200*, whereas *Glyma.16g018300* is located 1.9 kb downstream of AX-90496773. *Glyma.16g018200* encodes a protein whose family membership is unknown, although the homologous gene of *Arabidopsis thaliana* is *AT1g01080*. The product encoded by this gene belongs to the RNA-binding (RRM motif) protein family, which may participate in the post-transcriptional regulation of genes, including pre-mRNA splicing and the cellular localization and stability maintenance of RNA [50]. *Glyma.16g018300* is homologous to *AT1g01090*, and the proteins encoded by these genes share 80.3% amino acid sequence identity. *Glyma.16g018300* encodes the pyruvate dehydrogenase E1 component alpha subunit and may be involved in two pathways, PWY-5173 (acetyl-CoA biosynthesis) and PWY-5464 (cytosolic glycolysis, pyruvate dehydrogenase and TCA cycle). In *Arabidopsis thaliana*, the WRINKLED1 (WRI1) transcription factor plays a role of utmost importance during oil accumulation in maturing seeds, and *AT1g01090* is the putative target gene of WRI1 in the fatty acid synthesis pathway [51]. In addition, *Glyma.05g243400*, *Glyma.05g244100* and *Glyma.05g245300* are candidate genes for MCFS, and *Glyma.05g243400* and *Glyma.05g244100* are located 118 kb and 73 kb upstream of the large-effect marker AX-90460290, respectively. *Glyma.05g243400* is homologous to *AT1g1870*, which encodes a putative EF-1-α-related GTP-binding protein. The vacuole is an essential organelle for plant life and plays important roles in storage (ions, metabolites, and proteins), digestion, pH and ion homeostasis, turgor pressure maintenance, biotic and abiotic defense responses, toxic compound sequestration, and pigmentation [52]. Analysis of the vegetative vacuole proteome of *A. thaliana* predicted that *AT1g1870* may be related to vacuolar membrane fusion and remodeling [53]. *Glyma.05g244100* shares 83.2% amino acid sequence identity with *MOTHER OF FT AND TFL1 (MFT)*, which encodes a phosphatidylethanolamine-binding protein that regulates seed germination via the ABA and GA signaling pathways in *Arabidopsis thaliana* [54]. *Glyma.05g245300* is homologous to the *AT1g73660* gene, encoding a Raf-like MAPKKK. In *Arabidopsis thaliana*, the *AT1g73660*-encoded MAPKKK is a negative regulator of salt tolerance and may regulate targets involved in the salt stress response [55]. In the present study, the expression levels of the five abovementioned genes were significantly different between extreme materials during soybean seed development. Thus, we postulate that these five genes are candidate genes for PFW and MCFS. However, further evidence is needed to functionally validate this hypothesis.

## Conclusion

In this study, we identified 14, 15, 63 and 48 markers associated with PFW, SFW, SDW and MCFS, respectively, via GWAS. Most markers co-localized with previously reported yield-related QTLs. We were particularly interested in the large-effect markers AX-90496773 and AX-90460290, which had an impact on yield-related traits at the R6 stage. According to genetic annotation and expression analyses, a total of five putative genes, including *Glyma.16g018200*, *Glyma.16g018300 Glyma.05g243400, Glyma.05g244100* and *Glyma.05g245300,* are proposed as the candidate genes for PFW and MCFS, but further investigation is needed for verification of this hypothesis. These results provide insights into the yield improvement of vegetable soybean.

## Additional files

Additional file 1: **Table S1.** The ecological distribution of 133 soybean landraces. (XLSX 14 kb)

Additional file 2: **Table S2.** qRT-PCR primers. (XLSX 9 kb)

Additional file 3: **Table S3.** Summary of the polymorphic markers on the 20 chromosomes of *Glycine max*. (XLSX 10 kb)

Additional file 4: **Table S4.** The ecological distribution of 133 soybean landraces from different subgroups. (XLSX 9 kb)

Additional file 5: **Table S5.** SNPs significantly associated with the four yield-related traits. (XLSX 22 kb)

Additional file 6: **Figure S1.** Manhattan and quantile–quantile (QQ) plots of the GWAS for 100-pod fresh weight (PFW) in soybean at the R6 stage. The horizontal blue line indicates the genome-wide significance threshold (−log_10_(*P*) > 4.91); a, b and c represent 2015, 2016 and the means across the two years, respectively. (TIF 10418 kb)

Additional file 7: **Figure S2.** Manhattan and quantile–quantile (QQ) plots of the GWAS for 100-seed fresh weight (SFW) in soybean at the R6 stage. The horizontal blue line indicates the genome-wide significance threshold (−log_10_(*P*) > 4.91). a, b and c represent 2015, 2016 and the means across the two years, respectively. (TIF 5303 kb)

Additional file 8: **Figure S3.** Manhattan and quantile–quantile (QQ) plots of GWAS for 100-seed dry weight (SDW) in soybean at the R6 stage. The horizontal blue line indicates the genome-wide significance threshold (−log_10_(*P*) > 4.91). a, b and c represent 2015, 2016 and the means across the two years, respectively. (TIF 5874 kb)

Additional file 9: **Figure S4.** Manhattan and quantile–quantile (QQ) plots of GWAS for moisture content of fresh seeds (MCFS) in soybean at the R6 stage. The horizontal blue line indicates the genome-wide significance threshold (−log_10_(*P*) > 4.91). a, b and c represent 2015, 2016 and the means across the two years, respectively. (TIF 10454 kb)

## Abbreviations

ANOVA: Analysis of variance
GD: Gene diversity
GLM: A general linear model
GWAS: Genome-wide association study
*h*^*2*^: The broad-sense heritability
HHR: The Huanghuai region
LD: Linkage disequilibrium
MAFs: Minor allelic frequencies
MCFS: Moisture content of fresh seeds
MCMC: Monte Carlo Markov Chain
MLM: A mixed linear model
NER: The Northeast region
NJ: Neighbor-joining
NR: The North region
PCA: Principal component analysis
PFW: 100-pod fresh weight
PIC: Polymorphism information content
qRT-PCR: Quantitative real-time PCR
QTLs: Quantitative trait loci
*r*_*p*_: Phenotypic correlation coefficients
SDW: 100-seed dry weight
SFW: 100-seed fresh weight
SNPs: Single nucleotide polymorphisms
SR: The South region
