# Supplementary figures and images for: Genome-wide association study of four yield-related traits at the R6 stage in soybean
Source: BMC Genet. 2019 Mar 29;20:39. doi: 10.1186/s12863-019-0737-9 (PMC6440021; doi:10.1186/s12863-019-0737-9)

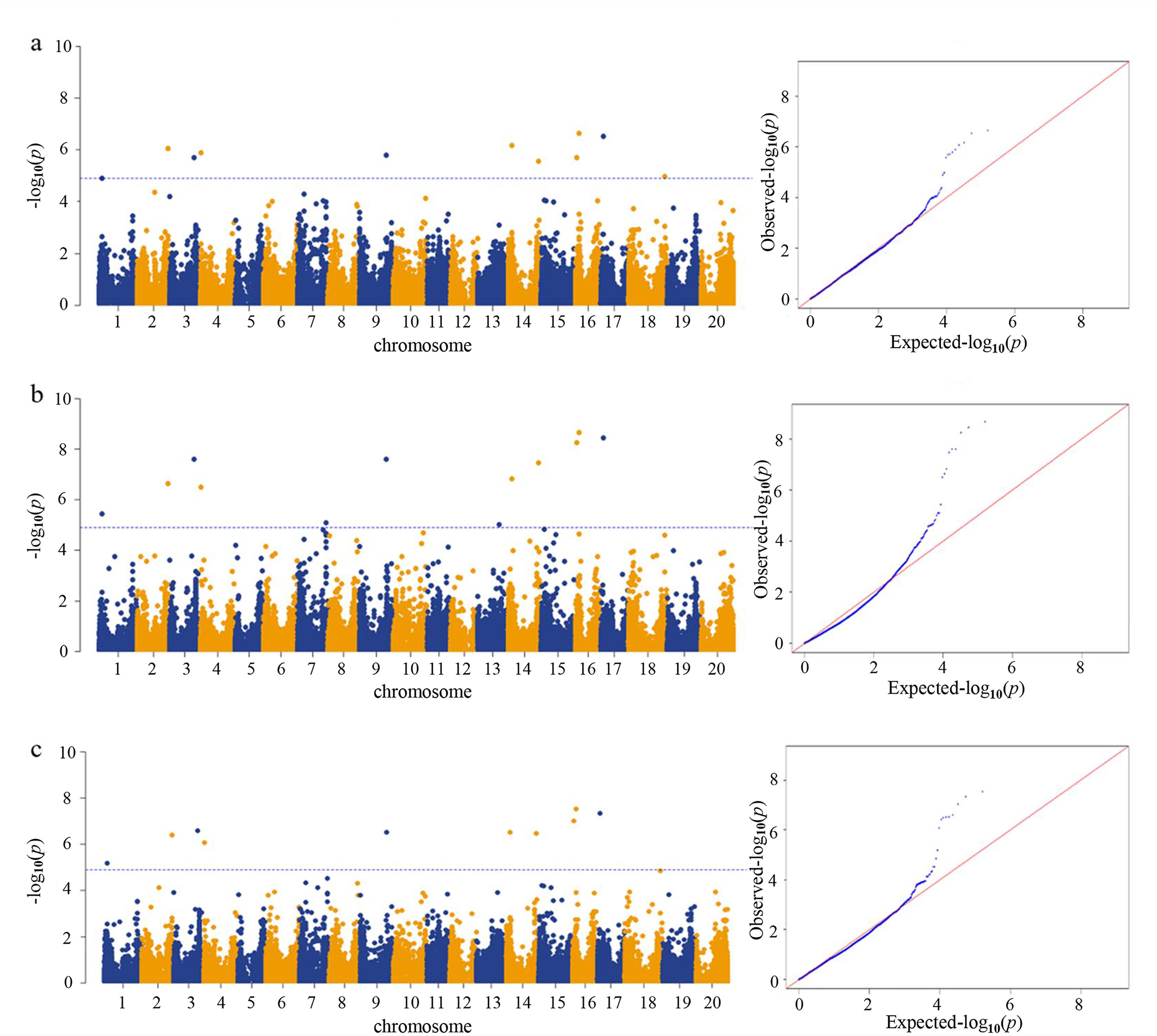

Supplement: Supplementary file 6 — Figure S1. Manhattan and quantile–quantile (QQ) plots of the GWAS for 100-pod fresh weight (PFW) in soybean at the R6 stage. The horizontal blue line indicates the genome-wide significance threshold (−log10(P) > 4.91); a, b and c represent 2015, 2016 and the means across the two years, respectively. (TIF 10418 kb) [file 12863_2019_737_MOESM6_ESM.tif]

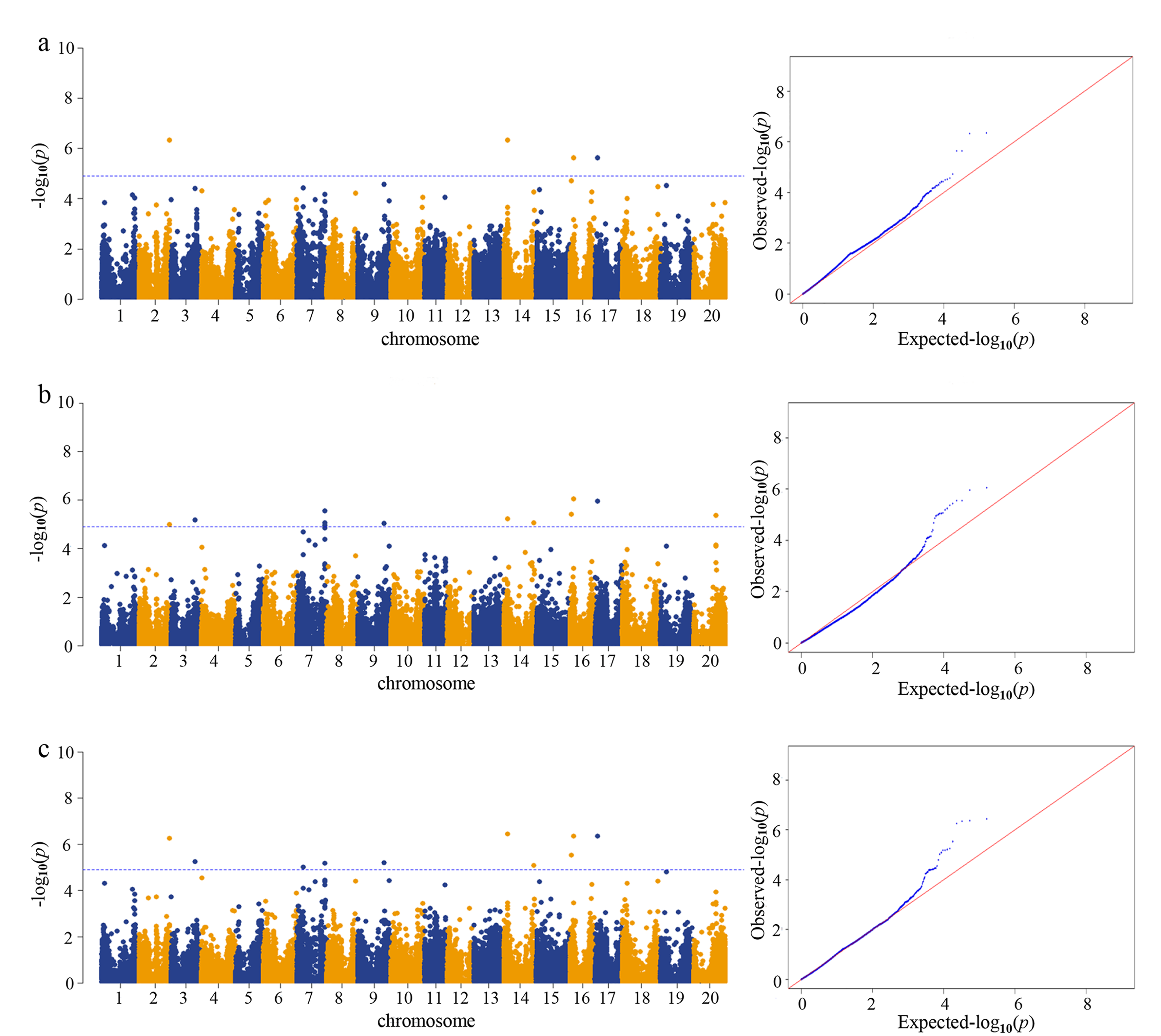

Supplement: Supplementary file 7 — Figure S2. Manhattan and quantile–quantile (QQ) plots of the GWAS for 100-seed fresh weight (SFW) in soybean at the R6 stage. The horizontal blue line indicates the genome-wide significance threshold (−log10(P) > 4.91). a, b and c represent 2015, 2016 and the means across the two years, respectively. (TIF 5303 kb) [file 12863_2019_737_MOESM7_ESM.tif]

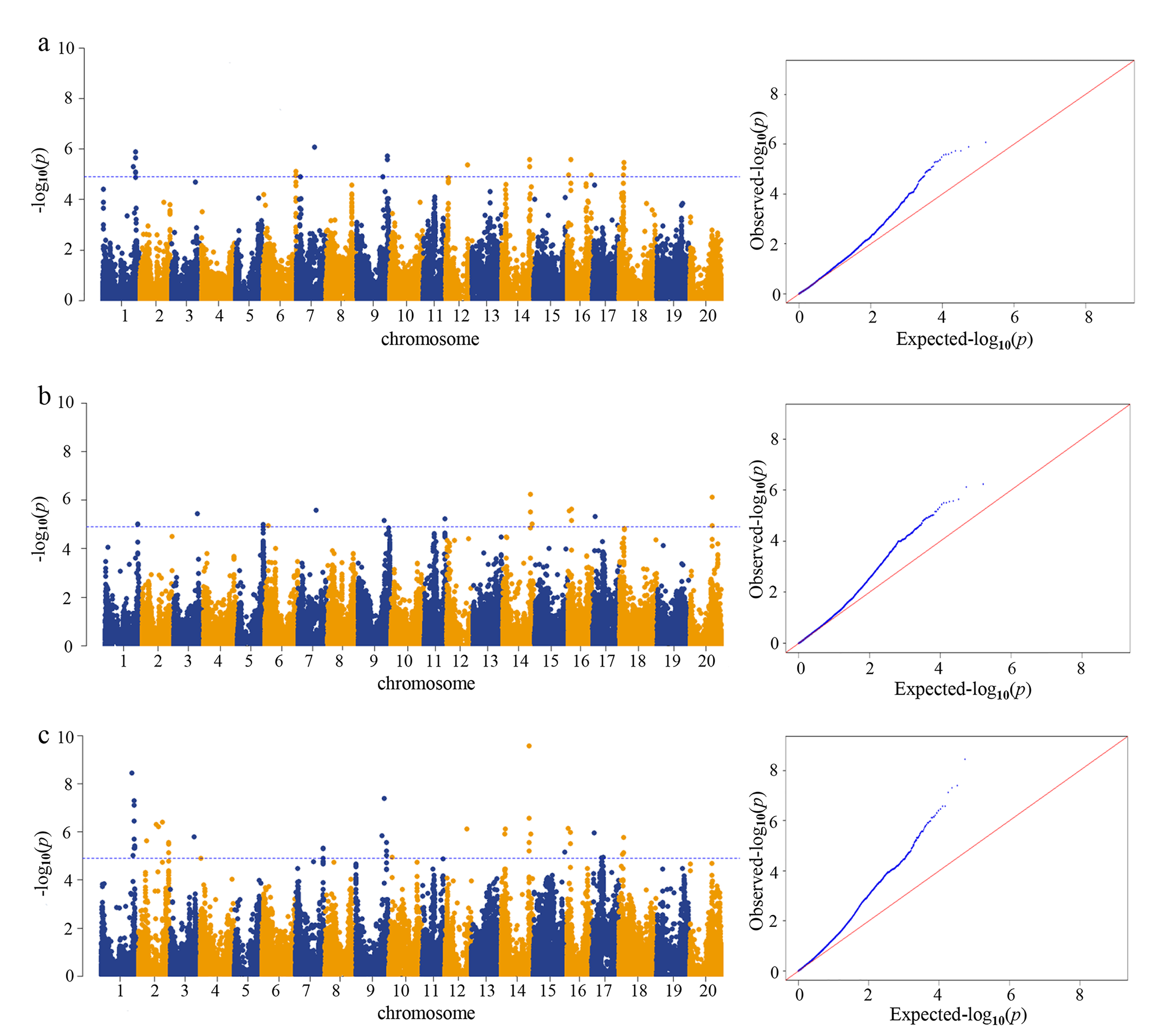

Supplement: Supplementary file 8 — Figure S3. Manhattan and quantile–quantile (QQ) plots of GWAS for 100-seed dry weight (SDW) in soybean at the R6 stage. The horizontal blue line indicates the genome-wide significance threshold (−log10(P) > 4.91). a, b and c represent 2015, 2016 and the means across the two years, respectively. (TIF 5874 kb) [file 12863_2019_737_MOESM8_ESM.tif]

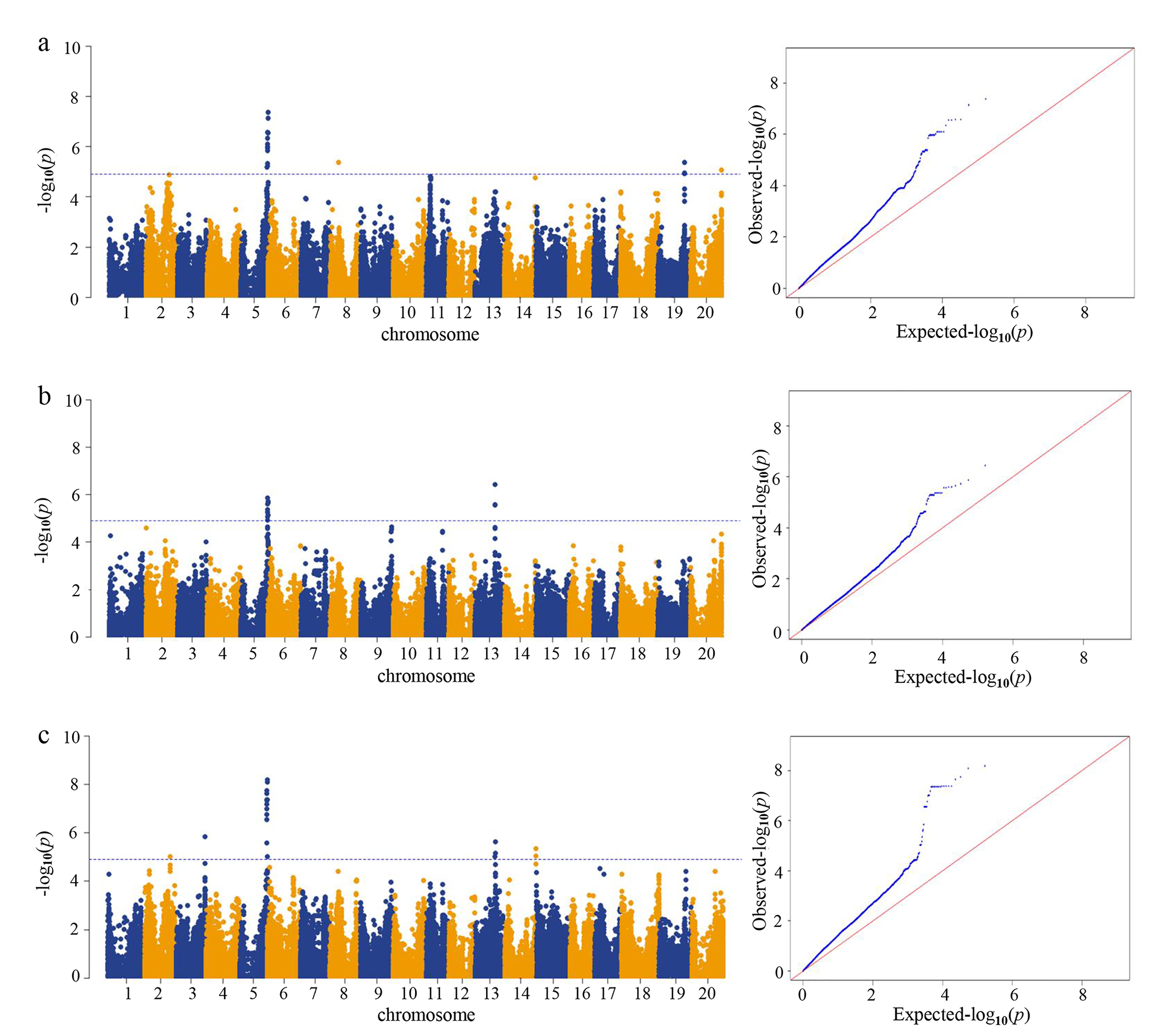

Supplement: Supplementary file 9 — Figure S4. Manhattan and quantile–quantile (QQ) plots of GWAS for moisture content of fresh seeds (MCFS) in soybean at the R6 stage. The horizontal blue line indicates the genome-wide significance threshold (−log10(P) > 4.91). a, b and c represent 2015, 2016 and the means across the two years, respectively. (TIF 10454 kb) [file 12863_2019_737_MOESM9_ESM.tif]
